# Excluding false negative error in certification of quantum channels

**DOI:** 10.1038/s41598-021-00444-x

**Published:** 2021-11-05

**Authors:** Aleksandra Krawiec, Łukasz Pawela, Zbigniew Puchała

**Affiliations:** 1 Institute of Theoretical and Applied Informatics, Polish Academy of Sciences, ul. Bałtycka 5, 44-100 Gliwice, Poland; 2 Faculty of Physics, Astronomy and Applied Computer Science, Jagiellonian University, ul. Łojasiewicza 11, 30-348 Kraków, Poland

**Keywords:** Quantum information, Quantum mechanics

## Abstract

Certification of quantum channels is based on quantum hypothesis testing and involves also preparation of an input state and choosing the final measurement. This work primarily focuses on the scenario when the false negative error cannot occur, even if it leads to the growth of the probability of false positive error. We establish a condition when it is possible to exclude false negative error after a finite number of queries to the quantum channel in parallel, and we provide an upper bound on the number of queries. On top of that, we found a class of channels which allow for excluding false negative error after a finite number of queries in parallel, but cannot be distinguished unambiguously. Moreover, it will be proved that parallel certification scheme is always sufficient, however the number of steps may be decreased by the use of adaptive scheme. Finally, we consider examples of certification of various classes of quantum channels and measurements.

## Introduction

Being deceived is not a nice experience. People have been developing plenty of methods to protect themselves against being cheated and one of these methods concerns verification of objects, also quantum ones. The cornerstone for theoretical studies on discrimination of quantum objects was laid by Helstrom^1^ a few decades ago.

In the era of Noisy Intermediate-Scale Quantum (NISQ) devices^2,3^, assuring the correctness of components in undeniably in the spotlight. A broad review of multipronged modern methods of certification as well as benchmarking of quantum states and processes can be found in the recent paper^4^. For a more introductory tutorial to the theory of system certification we refer the reader to^5^. Verification of quantum processes is often studied in the context of specific elements of quantum information processing tasks. Protocols for efficient certification of quantum processes, such as quantum gates and circuits, were recently studied in^6–8^.

Let us introduce the most general problem of verification studied in this work. Assume there are two known quantum channels and one of them is secretly chosen. Then, we are given the secretly chosen channel to verify which of the two channels it is. We are allowed to prepare any input state and apply the given channel on it. Finally, we can prepare any quantum measurement and measure the output state. Basing on the measurement’s outcome we make a decision which of the two channels was secretly chosen. In this work we focus on the case when we are promised which of the channels is given. After performing some certification procedure we can either agree that the channel was the promised one or claim that we were cheated. We want to assure that we will always realize when we are cheated. It may happen though, that we appear to be too suspicious and claim that we were cheated when we were not.

There are three major theoretical approaches towards verification of quantum channels called minimum error discrimination, unambiguous discrimination and certification. All these three approaches can be generalized to the multiple-shot case, that is when the given channel can be used multiple times in various configurations. The most straightforward possibility is the parallel scheme and the most sophisticated is the adaptive scheme (where we are allowed to use any processing between the uses of the given channel).

The first approach is called minimum error discrimination (a.k.a. distinguishability or symmetric discrimination) and makes use of the distance between quantum channels expressed by the use of the diamond norm. In this scenario one wants to minimize the probability of making the erroneous decision using the bound on this probability given by the Holevo-Helstrom theorem^1,9^. Single-shot discrimination of unitary channels and von Neumann measurements were studied in^10,11^ and^12–14^ respectively. Parallel discrimination of quantum channels was studied eg. in^15,16^. It appeared that parallel discrimination scheme is optimal in the case of distinguishability of unitary channels^17^ and von Neumann measurements^18^. In some cases however, the use of adaptive discrimination scheme can significantly improve the certification^19,20^. Advantages of the use of adaptive discrimination scheme in the asymptotic regime were studied in^21^. Fundamental and ultimate limits for quantum channel discrimination were derived in^22,23^. The works^24,25^ address the problem of distinguishability of quantum channels in the context of resource theory.

In the second approach, that is unambiguous discrimination, there are three possible outcomes. Two of them designate quantum channels while the third option is the inconclusive result. In this approach, when the result indicated which channel was given, we know it for sure. There is a chance however, that we will obtain an inconclusive answer. Unambiguous discrimination of quantum channels was considered in^26^, while unambiguous discrimination of von Neumann measurements was explored in^18^. Studies on unambiguous discrimination of quantum channels took a great advantage of unambiguous discrimination of quantum states, which can be found eg. in^27–31^.

The third approach, known as certification or asymmetric discrimination, is based on hypothesis testing. We are promised to be given one of the two channels and associate this channel with the null hypothesis, $H_0$. The other channel is associated with the alternative hypothesis, $H_1$. When making a decision whether to accept or to reject the null hypothesis, two types of errors may occur, that is we can come across false positive and false negative errors. In this work we consider the situation when we want to assure that false negative error will not occur, even if the probability of false positive error grows. A similar task of minimizing probability of false negative error having fixed bound on the probability of false positive was studied in the case of von Neumann measurements in^32^. Certification of quantum channels was studied in the asymptotic regime e.g. in^21,24,33^.

It should come as no surprise that in some cases perfect verification is not possible by any finite number of steps. Conditions for perfect minimum error discrimination of quantum operations were derived in^34^. Similar condition for unambiguous discrimination was proved in^26^. However, no such conditions have been stated for certification. In this work we derive a condition when we can exclude false negative error after a finite number of uses in parallel. This condition holds for arbitrary quantum channels and is expressed by the use of Kraus operators of these channels. We will provide an example of channels which can be certified in a finite number of queries in parallel, but cannot be distinguished unambiguously. Moreover, we will show that, in contrast to discrimination of quantum channels^19,20^, parallel certification scheme is always sufficient for certification, although the number of uses of the certified channel may not be optimal. On top of that, we will consider certification of quantum measurements and focus on the class of measurements with rank-one effects. The detailed derivation of the upper bound for the probability of false positive error will be presented for SIC POVMs.

This work is organized as follows. After introducing basic mathematical concepts in “Preliminaries” section, we present our main result, that is the condition when excluding false negative is possible in a finite number of uses in parallel, in Theorem 1 in “Parallel certification” section. Next, we apply this result to a specific subclass of quantum channels in “Certification of quantum measurements” section. Then, in “Adaptive certification and Stein setting” section we state, as Theorem 2,   the condition when excluding false negative error is possible in the adaptive scheme.   Finally, summary can be found in “Conclusions” section.

## Preliminaries

Let $\mathcal {D}_d$ denote the set of quantum states of dimension *d*, that is the set of positive semidefinite operators having trace equal one. Throughout this paper quantum states will be denoted by lower-case Greek letters, usually $\rho , \sigma , \tau$. For any state $\rho \in \mathcal {D}_d$ we can write its spectral decomposition as $\rho = \sum _i p_i |\lambda _i\rangle \! \langle \lambda _i|$. Having a set of quantum states $\{\rho _1, \ldots , \rho _m\}$ with spectral decompositions $\rho _1 = \sum _{i_1} p_{i_1} |\lambda _{i_1}\rangle \! \langle \lambda _{i_1}|, \ldots , \rho _m = \sum _{i_m} p_{i_m} |\lambda _{i_m}\rangle \! \langle \lambda _{i_m}|$ respectively, their *support* is defined as $\mathrm {supp}(\rho _1, \ldots , \rho _m) {:}{=}\mathrm {span}\{ |\lambda _{i_j}\rangle : p_{i_j} > 0 \}$. The set of unitary matrices of dimension *d* will be denoted $\mathcal {U}_d$.

Quantum channels are linear maps which are completely positive and trace preserving. In this work we will often take advantage of the Kraus representations of channels. Let

1 $$\begin{aligned} \Phi _0(X) := \sum _{i=1}^k E_i X E_i^\dagger , \quad \Phi _1(X) := \sum _{j=1}^l F_j X F_j^\dagger \end{aligned}$$

be the Kraus representations of the channels that will correspond to null and alternative hypotheses respectively. The sets of operators $\{E_i\}_i$ and $\{F_j\}_j$ are called Kraus operators of channels $\Phi _0$ and $\Phi _1$ respectively. We will use the notation $\mathrm {supp}(\Phi _0) {:}{=}\mathrm {span}\{E_i \}_i$, $\mathrm {supp}(\Phi _1) {:}{=}\mathrm {span}\{F_j \}_j$, to denote the supports of quantum channels. Moreover, the notation ${\mathbbm{1}}$ will be used for the identity channel.

The most general quantum measurements, known also as POVMs (positive operator valued measure) are defined as a collection of positive semidefinite operators $\mathcal {P}= \{ M_1, \ldots , M_m \}$ which fulfills the condition $\sum _{i=1}^m M_i = {\mathbbm{1}} _{d}$, where ${\mathbbm{1}} _{d}$ denotes the identity matrix of dimension *d*. When a quantum state $\rho$ is measured by the measurement $\mathcal {P}$, then the label *i* is obtained with probability ${{\,\mathrm{Tr}\,}}(E_i \rho )$ and the state $\rho$ ceases to exist. A special class of quantum measurements are projective von Neumann measurements. These POVMs have rank-one effects of the form $\{ |u_1\rangle \! \langle u_1|, \ldots , |u_d\rangle \! \langle u_d| \}$, where vectors $\{|u_i\rangle \}_{i=1}^d$ form an orthonormal basis and therefore they are columns of some unitary matrix $U \in \mathcal {U}_d$.

Now we proceed to describing the detailed scheme of certification. There are two quantum channels: $\Phi _0$ and $\Phi _1$. We are promised that we are given $\Phi _0$ but we are not sure and we want to verify it using hypothesis testing. We associate the channel $\Phi _0$ with the null hypothesis $H_0$ and we associate the other channel $\Phi _1$ with the alternative hypothesis $H_1$. We consider the following scheme. We are allowed to prepare any (possibly entangled) input state and perform the given channel on it. Then, we prepare a binary measurement $\{\Omega _0, {\mathbbm{1}} - \Omega _0\}$ and measure the output state. If we obtain the label associated with the effect $\Omega _0$, then we decide that the certified channel was $\Phi _0$ and we accept the null hypothesis. If we get the label associated with the effect ${\mathbbm{1}} - \Omega _0$, then we decide that the certified channel was $\Phi _1$ and therefore we reject the null hypothesis.

The aim of certification is to make a decision whether to accept or to reject $H_0$. While making such a decision one can come upon two types of errors. The false positive error (also known as type I error) happens when we reject the null hypothesis when in fact it was true. The converse situation, that is accepting the null hypothesis when the alternative hypothesis was correct, is known as the false negative (or type II) error. In this work we will focus on the situation when the probability of the false negative error equals zero and we want to minimize the probability of false positive error.

Let us now take a closer look into the scheme of entanglement-assisted single-shot certification procedure. We begin with preparing an input state $|\psi \rangle$ on the compound space. Then, we apply the certified channel extended by the identity channel on the input state, obtaining as the output the state either $\rho _0^{|\psi \rangle } = \left( \Phi _0 \otimes {\mathbbm{1}} \right) (|\psi \rangle \! \langle \psi |)$, if the given channel was $\Phi _0$, or $\rho _1^{|\psi \rangle } = \left( \Phi _1 \otimes {\mathbbm{1}} \right) (|\psi \rangle \! \langle \psi |)$, if the given channel was $\Phi _1$. Eventually, we perform the measurement $\{\Omega _0, {\mathbbm{1}} - \Omega _0 \}$, where the effect $\Omega _0$ accepts hypothesis $H_0$ and the effect ${\mathbbm{1}} -\Omega _0$ accepts the alternative hypothesis $H_1$.

Assuming that the input state $|\psi \rangle$ and measurement effect $\Omega _0$ have been fixed, the probability of making the false positive error is given by

2 $$\begin{aligned} p_1 \left( |\psi \rangle , \Omega _0 \right) {:}{=}{{\,\mathrm{Tr}\,}}\left( ({\mathbbm{1}} - \Omega _0) \rho _0^{|\psi \rangle } \right) = 1- {{\,\mathrm{Tr}\,}}\left( \Omega _0 \rho _0^{|\psi \rangle } \right) . \end{aligned}$$

In a similar manner we have the probability of making the false negative error, that is

3 $$\begin{aligned} p_2 \left( |\psi \rangle , \Omega _0 \right) {:}{=}{{\,\mathrm{Tr}\,}}\left( \Omega _0 \rho _1^{|\psi \rangle } \right) . \end{aligned}$$

We will be interested in the situation when probability of the false negative error is equal to zero and we want to minimize the probability of false positive error. Therefore, we introduce the notation

4 $$\begin{aligned} p_1 {:}{=}\min _{|\psi \rangle , \Omega _0} \left\{ p_1 \left( |\psi \rangle , \Omega _0 \right) : \ p_2 \left( |\psi \rangle , \Omega _0 \right) = 0 \right\} \end{aligned}$$

for minimized probability of false positive error in the single-shot scenario.

For a given $\epsilon >0$, we say that quantum channel $\Phi _0$ *can be* $\epsilon$-*certified against* channel $\Phi _1$ if there exist an input state $|\psi \rangle$ and measurement effect $\Omega _0$ such that $p_2 \left( |\psi \rangle , \Omega _0 \right) = 0$ and $p_1 \left( |\psi \rangle , \Omega _0 \right) \le \epsilon$. In other words, quantum channel $\Phi _0$ can be $\epsilon$-certified against another channel $\Phi _1$ if we can assure no false negative will occur and the probability of false positive error is smaller than $\epsilon$.

When performing the certification of quantum channels, we can use the channels many times in various configurations. Now we proceed to introducing notation needed for studying parallel and adaptive certification schemes.

### Parallel certification scheme

**Figure 1:**
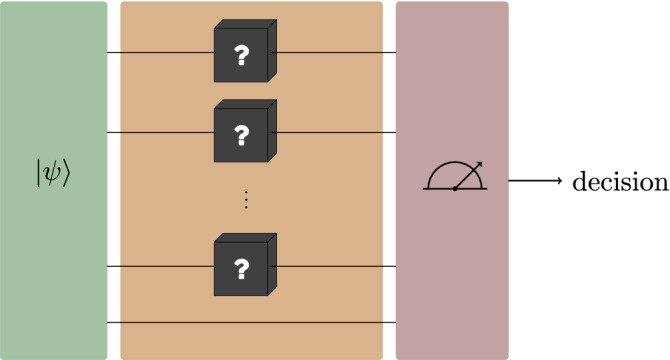
Parallel certification scheme.

Let *N* denote the number of uses of the quantum channel in parallel. A schematic representation of the scenario of parallel certification is depicted in Fig. 1. In this scheme we consider certifying tensor products of the channels. In other words, parallel certification of channels $\Phi _0$ and $\Phi _1$ can be seen as certifying channels $\Phi _0^{\otimes N}$ and $\Phi _1^{\otimes N}$ for some natural number *N*.

Let $|\psi \rangle$ be the input state to the certification procedure. After applying the channel $\Phi _0$ *N* times in parallel, we obtain the output state

5 $$\begin{aligned} \sigma _0^{N, |\psi \rangle } = \left( \Phi _0^{\otimes N} \otimes {\mathbbm{1}} \right) (|\psi \rangle \! \langle \psi |), \end{aligned}$$

if the channel was $\Phi _0$, and similarly

6 $$\begin{aligned} \sigma _1^{N, |\psi \rangle } = \left( \Phi _1^{\otimes N} \otimes {\mathbbm{1}} \right) (|\psi \rangle \! \langle \psi |), \end{aligned}$$

if the channel was $\Phi _1$. In the same spirit let

7 $$\begin{aligned} p_1^{\mathbb{P} , N} \left( |\psi \rangle , \Omega _0 \right) = {{\,\mathrm{Tr}\,}}\left(({\mathbbm{1}} - \Omega _0) \sigma _0^{N, |\psi \rangle }\right) , \quad p_2^{\mathbb{P} , N} \left( |\psi \rangle , \Omega _0 \right) = {{\,\mathrm{Tr}\,}}\left(\Omega _0 \sigma _1^{N, |\psi \rangle } \right) \end{aligned}$$

be the probabilities of occurring false positive and false negative errors respectively. When $N=1$, then we arrive at single-shot certification. Therefore we will neglect the upper index and simply write $p_1 \left( |\psi \rangle , \Omega _0 \right)$ and $p_2 \left( |\psi \rangle , \Omega _0 \right)$.

We introduce the notation

8 $$\begin{aligned} p_1^{\mathbb{P} , N} {:}{=}\min _{|\psi \rangle , \Omega _0} \left\{ p_1^{\mathbb{P} , N} \left( |\psi \rangle , \Omega _0 \right) : \ p_2^{\mathbb{P} , N} \left( |\psi \rangle , \Omega _0 \right) = 0 \right\} \end{aligned}$$

for the minimized probability of false positive error in the parallel scheme.

We say that quantum channel $\Phi _0$ *can be certified against* $\Phi _1$ *in the parallel scheme*, if for every $\epsilon >0$ there exist a natural number *N*, an input state $|\psi \rangle$ and measurement effect $\Omega _0$ such that $p_2^{\mathbb{P}, N} \left( |\psi \rangle , \Omega _0 \right) = 0$ and $p_1^{\mathbb{P}, N} \left( |\psi \rangle , \Omega _0\right) \le \epsilon$.

Let us now elaborate a bit on the number of steps needed for certification. Assume that we have fixed upper bound on the probability of false positive error, $\epsilon >0$. We will be interested in calculating the minimal number of queries, $N_\epsilon$, for which $p_2^{\mathbb{P}, N} \left( |\psi \rangle , \Omega _0 \right) = 0$ and $p_1^{\mathbb{P}, N} \left( |\psi \rangle , \Omega _0\right) \le \epsilon$ for some input state $|\psi \rangle$ and measurement effect $\Omega _0$. Such a number, $N_\epsilon$, will be called the *minimal number of steps needed for parallel certification*.

### Adaptive certification scheme

Adaptive certification scheme allows for the use of processing between the uses of the certified channel, therefore this procedure is more complex then the parallel certification. However, when the processings only swap the subsystems, then the adaptive scheme may reduce to the parallel one.

**Figure 2:**
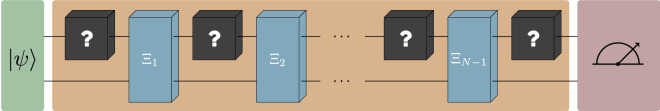
Adaptive certification scheme. The processings $\Xi _1, \ldots , \Xi _{N-1}$ can be arbitrary quantum channels where we only assume that the first subsystem must fit the input of the black box. In particular, the processings can swap subsystems, and therefore one can obtain the parallel scheme as a special case of the adaptive discrimination scheme.

Assume as previously that $|\psi \rangle$ is the input state to the certification procedure in which the certified channel in used *N* times and any processing is allowed between the uses of this channel. The scheme of this procedure is presented in the Fig. 2. Having the input state $|\psi \rangle$ on the compound register, we perform the certified channel (denoted by the black box with question mark) on one part of it. Having the output state we can perform some processing $\Xi _1$ and therefore get prepared for the next use of the certified channel. Than again, we apply the certified channel on one register of the prepared state and again, we can perform processing $\Xi _2$. We repeat this procedure $N-1$ times. After the *N*-th use of the certified channel we obtain the state either $\tau _0^{N, |\psi \rangle }$, if the channel was $\Phi _0$, or $\tau _1^{N, |\psi \rangle }$, if the channel was $\Phi _1$. Then, we prepare a global measurement $\{\Omega _0, {\mathbbm{1}} - \Omega _0\}$ and apply it on the output state. Let

9 $$\begin{aligned} p_1^{\mathbb{A} , N} \left( |\psi \rangle , \Omega _0 \right) = {{\,\mathrm{Tr}\,}}\left(({\mathbbm{1}} - \Omega _0) \tau _0^{N, |\psi \rangle }\right) , \quad p_2^{\mathbb{A} , N} \left( |\psi \rangle , \Omega _0 \right) = {{\,\mathrm{Tr}\,}}\left(\Omega _0 \tau _1^{N, |\psi \rangle } \right) \end{aligned}$$

be the probabilities of the false positive and false negative errors in adaptive scheme, respectively, when the input state and the measurement effects were fixed. When $N=1$, then we will neglect the upper index and simply write $p_1 \left( |\psi \rangle , \Omega _0 \right)$ and $p_2 \left( |\psi \rangle , \Omega _0 \right)$.

We say that quantum channel $\Phi _0$ *can be certified against* $\Phi _1$ *in the adaptive scheme*, if for every $\epsilon >0$ there exist a natural number *N*, an input state $|\psi \rangle$ and measurement effect $\Omega _0$ such that $p_2^{\mathbb{A} , N} \left( |\psi \rangle , \Omega _0 \right) = 0$ and $p_1^{\mathbb{A} , N} \left( |\psi \rangle , \Omega _0\right) \le \epsilon$.

For a fixed upper bound on the probability of false positive error, $\epsilon$, we introduce the *minimal number of steps needed for adaptive certification*, $N_\epsilon$, as the minimal number of steps after which $p_2^{\mathbb{A} , N} \left( |\psi \rangle , \Omega _0 \right) = 0$ and $p_1^{\mathbb{A} , N} \left( |\psi \rangle , \Omega _0\right) \le \epsilon$ for some input state $|\psi \rangle$ and measurement effect $\Omega _0$.

## Parallel certification

Not all quantum channels can be discriminated perfectly after a finite number of queries. Conditions for perfect discrimination were states in the work^34^. Similar conditions for unambiguous discrimination were proved in^26^. In this section we will complement these results with the condition concerning parallel certification. More specifically, we will prove a simple necessary and sufficient condition when a quantum channel $\Phi _0$ can be certified against some other channel $\Phi _1$. As the condition utilizes the notion of the support of a quantum channel, recall that it is defined as the span of their Kraus operators. The condition will be stated as Theorem 1, however its proof will be presented after introducing two technical lemmas.

In fact, the statement of Theorem 1 is a bit more general, that is it concerns the situation when the alternative hypothesis corresponds to a set of channels $\left\{ \Phi _1, \ldots , \Phi _m \right\}$ having Kraus operators $\left\{ F^{(1)}_{{j_1}} \right\} _{{j_1}}, \ldots , \left\{ F^{(m)}_{{j_m}} \right\} _{{j_m}}$ respectively. We will use the notation $\mathrm {supp}\left( \Phi _1, \ldots , \Phi _m \right) {:}{=}\mathrm {span}\left\{ F^{(1)}_{{j_1}} , \ldots , F^{(m)}_{{j_m}} \right\} _{ {j_1}, \ldots ,{j_m}}$.

### Theorem 1

Quantum channel $\Phi _0$ can be certified against quantum channels $\Phi _1, \ldots , \Phi _m$ in the parallel scheme if and only if $\mathrm {supp}(\Phi _0) \not \subseteq \mathrm {supp}\left( \Phi _1, \ldots , \Phi _m \right)$.

Moreover, to ensure that the probability of false positive error is no greater than $\epsilon$, the number of steps needed for parallel certification is bounded by $N_\epsilon \ge \left\lceil \frac{\log \epsilon }{\log p_1}\right\rceil$, where $p_1$ is the upper bound on probability of false positive error in single-shot certification.

Before presenting the proof of this theorem we will introduce two lemmas. The proofs of lemmas are postponed to “Supplementary Appendix A”. Lemma 1 states that if the inclusion does not hold for supports of the quantum channels, then the inclusion also does not hold for supports of output states assuming that the input state has full Schmidt rank. The proof of Lemma 1 is based on the proof in^26^[Theorem  1], which studies unambiguous discrimination among quantum operations.

### Lemma 1

Let $\{|a_t\rangle \}_t$ and $\{|b_t\rangle \}_t$ be two orthonormal bases and $|\psi \rangle {:}{=}\sum _t \lambda _t |a_t\rangle |b_t\rangle$ where $\lambda _t>0$ for every *t*. Let also $\rho _0^{|\psi \rangle } = \left( \Phi _0 \otimes {\mathbbm{1}} \right) (|\psi \rangle \! \langle \psi |)$ and $\rho _j^{|\psi \rangle } = \left( \Phi _j \otimes {\mathbbm{1}} \right) (|\psi \rangle \! \langle \psi |)$ for $j=1, \ldots , m$. If $\mathrm {supp}(\Phi _0) \not \subseteq \mathrm {supp}(\Phi _1, \ldots, \Phi _m)$, then $\mathrm {supp}\left( \rho _0^{|\psi \rangle }\right) \not \subseteq \mathrm {supp}\left( \rho _1^{|\psi \rangle } , \ldots , \rho _m^{|\psi \rangle }\right)$.

Lemma 2 also concerns inclusions of supports. It states that if the inclusion of supports does not hold for some output states, then it does not hold also for supports of the channels.

### Lemma 2

With the notation as above, if there exists a natural number *N* and an input state $|\psi \rangle$ such that $\mathrm {supp}\left( \sigma _0^{N, |\psi \rangle } \right) \not \subseteq \mathrm {supp}\left( \sigma _1^{N, |\psi \rangle }, \ldots , \sigma _m^{N, |\psi \rangle } \right)$, then $\mathrm {supp}(\Phi _0) \not \subseteq \mathrm {supp}\left( \Phi _1, \ldots ,\Phi _m\right)$.

Finally, we are in position to present the proof of Theorem 1.

### Proof of Theorem 1

(⇐) Let $\mathrm {supp}(\Phi _0) \not \subseteq \mathrm {supp}\left( \Phi _1, \ldots , \Phi _m \right)$. From Lemma 1 this implies $\mathrm {supp}\left( \rho _0^{|\psi \rangle } \right) \not \subseteq \mathrm {supp}\left( \rho _1^{|\psi \rangle } , \ldots , \rho _m^{|\psi \rangle }\right)$ where the input state is $|\psi \rangle =\sum _t \lambda _t |a_t\rangle |b_t\rangle$. Hence we can always find a state $|\phi _0\rangle$ for which

10 $$\begin{aligned} |\phi _0\rangle \not \perp \mathrm {supp}\left( \rho _0^{|\psi \rangle } \right) \quad \text {and} \quad |\phi _0\rangle \perp \mathrm {supp}\left( \rho _1^{|\psi \rangle } , \ldots , \rho _m^{|\psi \rangle }\right) , \end{aligned}$$

and therefore

11 $$\begin{aligned} \langle \phi _0|\rho _0^{|\psi \rangle } |\phi _0\rangle >0 \quad \text {and} \quad \langle \phi _0|\rho _i^{|\psi \rangle } |\phi _0\rangle = 0 \end{aligned}$$

for $$i = 1 , \ldots , m$$.

Now we consider the certification scheme by taking the measurement with effects $\{\Omega _0, {\mathbbm{1}} - \Omega _0 \}$. Without loss of generality we can assume that $\Omega _0 {:}{=}|\phi _0\rangle \! \langle \phi _0|$ is a rank-one operator. We calculate

12 $$\begin{aligned} &{{\,\mathrm{tr}\,}}\left( \Omega _0 \rho _0^{|\psi \rangle } \right) = \langle \phi _0| \rho _0^{|\psi \rangle } |\phi _0\rangle >0 \\&p_2 \left( |\psi \rangle , \Omega _0 \right) = \sum _{i=1}^{m} {{\,\mathrm{tr}\,}}\left( \Omega _0 \rho _i^{|\psi \rangle } \right) = \sum _{i=1}^{m} \langle \phi _0| \rho _i^{|\psi \rangle } |\phi _0\rangle =0 \\&p_1 \left( |\psi \rangle , \Omega _0 \right) = {{\,\mathrm{tr}\,}}\left( ({\mathbbm{1}} - \Omega _0) \rho _0^{|\psi \rangle } \right) = 1- \langle \phi _0| \rho _0^{|\psi \rangle } |\phi _0\rangle < 1. \end{aligned}$$

Hence after sufficiently many uses, *N*, of the certified channel in parallel (actually when $N \ge \left\lceil \frac{\log \epsilon }{\log p_1}\right\rceil$) we obtain that ${{\,\mathrm{tr}\,}}\left( \Omega _1^{\otimes N} {\left( \rho _0^{|\psi \rangle }\right) }^{\otimes N} \right) \le \epsilon$ for any positive $\epsilon$. Therefore after *N* queries we will be able to exclude false negative error.

($\implies$) Assume that $\Phi _0$ can be certified against $\Phi _1, \ldots , \Phi _m$ in the parallel scenario. This means that there exist a natural number *N*, an input state $|\psi \rangle$ and a positive operator (measurement effect) $\Omega _0$ on the composite system such that

13 $$\begin{aligned}&p_1^{\mathbb{P}, N} \left( |\psi \rangle , \Omega _0\right) = 1- {{\,\mathrm{tr}\,}}\left( \Omega _0 \left( \Phi _0^{\otimes N} \otimes {\mathbbm{1}} \right) ( |\psi \rangle \! \langle \psi |)\right) \le \epsilon <1 \\&p_2^{\mathbb{P}, N} \left( |\psi \rangle , \Omega _0\right) = \sum _{i=1}^{m} {{\,\mathrm{tr}\,}}\left( \Omega _0 \left( \Phi _i^{\otimes N} \otimes {\mathbbm{1}} \right) ( |\psi \rangle \! \langle \psi |)\right) =0. \end{aligned}$$

Therefore ${{\,\mathrm{tr}\,}}\left( \Omega _0 \left( \Phi _0^{\otimes N} \otimes {\mathbbm{1}} \right) ( |\psi \rangle \! \langle \psi |)\right) >0$ and thus

14 $$\begin{aligned} \Omega _0& \perp \mathrm {supp}\left( \left( \Phi _0^{\otimes N} \otimes {\mathbbm{1}} \right) \left( |\psi \rangle \! \langle \psi |\right) \right) = \mathrm {span}\left\{ \left( E_{i_1} \otimes \ldots \otimes E_{i_N} \otimes {\mathbbm{1}} \right) |\psi \rangle \right\} _{i_1, \ldots, i_N} \\ \Omega _0&\perp \mathrm {supp}\left( \left( \Phi _1^{\otimes N} \otimes {\mathbbm{1}} \right) (|\psi \rangle \! \langle \psi |), \ldots , \left( \Phi _m^{\otimes N} \otimes {\mathbbm{1}} \right) (|\psi \rangle \! \langle \psi |) \right) \\&= \mathrm {span}\left\{ \left( K_{l_1} \otimes \ldots \otimes K_{l_N} \otimes {\mathbbm{1}} \right) |\psi \rangle \right\} _{l_1, \ldots, l_N}, \end{aligned}$$

where $$\mathrm {span}\left\{ F^{(1)}_{{j_1}} , \ldots , F^{(m)}_{{j_m}} \right\} _{ {j_1}, \ldots ,{j_m}} = \mathrm {span}\{K_l\}_l$$.

Hence

15 $$\begin{aligned}  \mathrm {span}\left\{ \left({\mathrm {E}}_{i_1} \otimes \ldots \otimes \text {E}_{i_\text {N}} \otimes {\mathbbm{1}} \right) |\psi \rangle \right\} _{i_1, \ldots, i_N} \not \subseteq \mathrm {span}\left\{ \left( {\text {K}}_{l_1} \otimes \ldots \otimes \text {K}_{l_\text {N}} \otimes {\mathbbm{1}} \right) |\psi \rangle \right\} _{l_1, \ldots, l_\text {N}}. \end{aligned}$$

The reminder of the proof follows directly from Lemma 2. $\square$

It is worth mentioning that in the above proof the measurement effect $\Omega _0$ is a rank-one projection operator. This is sufficient to prove that quantum channel $\Phi _0$ can be certified against $\Phi _1$ in the parallel scheme, but this is, in most of the cases, not optimal.

In the remaining of this section we will discuss two examples. The first example shows that if quantum channels can be certified in the parallel scheme, then it does not have to imply that they can be discriminated unambiguously. We will provide an explicit example of mixed-unitary channels which fulfill the condition from Theorem 1, and therefore can be certified in the parallel scheme, but cannot be discriminated unambiguously. In the second example we will consider the situation when the channel associated with the $H_1$ hypothesis is the identity channel and derive an upper bound on the probability of false positive error.

### Channels which cannot be discriminated unambiguously but still can be certified

In this subsection we will give an example of a class of channels which cannot be discriminated unambiguously, but they can be certified by a finite number of uses in the parallel scheme. The work^26^ presents the condition when quantum channels can be unambiguously discriminated by a finite number of uses. More precisely, Theorem 2 therein states that if a set of quantum channels $\mathcal {S} = \{\Phi _i\}_i$ satisfies the condition $\mathrm {supp}(\Phi _i) \not \subseteq \mathrm {supp}(\Phi _j)$ for every $\Phi _i, \Phi _j \in \mathcal {S}$, then they can be discriminated unambiguously in a finite number of uses.

Now we proceed to presenting our example. Let $\Phi _0$ be a mixed unitary channel of the form

16 $$\begin{aligned} \Phi _0(\rho ) = \sum _{i=1}^m p_i U_i \rho U_i^\dagger , \end{aligned}$$

where $p = (p_1, \ldots , p_m)$ is a probability vector and $\{ U_1, \ldots , U_m\}$ are unitary matrices. As the second channel we take a unitary channel of the form $\Phi _1(\rho ) = \tilde{U} \rho \tilde{U}^\dagger$, where we make a crucial assumption that $\tilde{U} \in \{ U_1, \ldots , U_m\}$.

Therefore we have $\mathrm {supp}(\Phi _0) = \mathrm {span}\{\sqrt{p_i}U_i \}_i$, while ${\mathrm{supp}}(\Phi _1) = {\mathrm {span}}\{\tilde{U}\}$. In this example it can be easily seen that the condition for unambiguous discrimination is not fulfilled as $\mathrm {supp}(\Phi _1) \subseteq \mathrm {supp}(\Phi _0)$. Nevertheless, the condition from Theorem 1 is fulfilled as $\mathrm {supp}(\Phi _0) \not \subseteq \mathrm {supp}(\Phi _1)$, and hence it is possible to exclude false negative error after a finite number of queries in parallel.

### Certification of arbitrary channel against the identity channel

Assume that we want to certify channel $\Phi _0$, which Kraus operators are $\{E_i\}_i$, against the identity channel $\Phi _1$ having Kraus operator $\{{\mathbbm{1}} \}$. We will show that as long as the channel $\Phi _0$ is not the identity channel, it can always be certified against the identity channel in the parallel scheme.

#### Proposition 1

Every quantum channel (except the identity channel) can be certified against the identity channel in the parallel scheme.

#### Proof

Let $|\psi \rangle$ be an input state. After applying the certified channels on it, we obtain the state either $\rho _0^{|\psi \rangle } = \left( \Phi _0 \otimes {\mathbbm{1}} \right) \left( |\psi \rangle \! \langle \psi |\right)$, if the channel was $\Phi _0$, or $\rho _1^{|\psi \rangle } = |\psi \rangle \! \langle \psi |$, if the channels was $\Phi _1$. As the final measurement effect we can take $\Omega _0 {:}{=}{\mathbbm{1}} - |\psi \rangle \! \langle \psi |$, which is always orthogonal to $\rho _1^{|\psi \rangle }$, hence no false negative error will occur. Having the input state and final measurement fixed, we will calculate the probability of false positive error in the single-shot scheme

17 $$\begin{aligned} p_1 (|\psi \rangle , \Omega _0)&= 1- {{\,\mathrm{Tr}\,}}\left( \Omega _0 \rho _0^{|\psi \rangle } \right) = 1-{{\,\mathrm{Tr}\,}}\left( \left( {\mathbbm{1}} - |\psi \rangle \! \langle \psi |\right) \rho _0^{|\psi \rangle } \right) ={{\,\mathrm{Tr}\,}}\left( |\psi \rangle \! \langle \psi | \rho _0^{|\psi \rangle } \right) \\&= \langle \psi | \left( \left( \Phi _0\otimes {\mathbbm{1}} \right) (|\psi \rangle \! \langle \psi |) \right) |\psi \rangle <1, \end{aligned}$$

where the last inequality follows from the fact that $\Phi _0$ is not the identity channel. Therefore, after sufficiently many queries in the parallel scheme the probability of false positive error will be arbitrarily small. $\square$

Note that the expression for the probability of false positive error in Eq. (17) is in fact the fidelity between the input state and the output of the channel $\Phi _0$ extended by the identity channel. As we were not imposing any specific assumptions on the input state, we can take the one which minimizes the expression in Eq. (17). Therefore, the probability of the false positive error in the single-shot certification yields

18 $$\begin{aligned} p_1 = \min _{|\psi \rangle } \langle \psi | \left( \left( \Phi _0\otimes {\mathbbm{1}} \right) (|\psi \rangle \! \langle \psi |) \right) |\psi \rangle . \end{aligned}$$

Eventually, to make sure that the probability of false positive error will not be greater than $\epsilon$, we will need $N_\epsilon \ge \left\lceil \frac{\log \epsilon }{\log p_1} \right\rceil$ steps in the parallel scheme.

From the above considerations we can draw a simple conclusion concerning the situation when $\Phi _0(X)= UX U^\dagger$ is a unitary channel. Then, as the unitary channel has only one Kraus operator, it holds that $p_1 =\min _{|\psi \rangle } \left| \langle \psi | \left( U \otimes {\mathbbm{1}} \right) |\psi \rangle \right| ^2 = \min _{|\psi \rangle } \left| \langle \psi | U |\psi \rangle \right| ^2 = \nu ^2(U)$, where $\nu (U)$ is the distance from zero to the numerical range of the matrix *U*^18,32^. Thanks to this geometrical representation (see further^18^) one can deduce the connection between the probability of false positive error, $p_1$, and the probability of making an error in the unambiguous discrimination of unitary channels. More specifically, let $p^u_{\mathrm {error}}$ denote the probability of making an erroneous decision in unambiguous discrimination of unitary channels. Then, it holds that $p^u_{\mathrm {error}} = p_1^2$. Therefore, in the case of certification of unitary channels the probability of making the false positive error is significantly smaller than the probability of erroneous unambiguous discrimination.

## Certification of quantum measurements

In this section we will take a closer look into the certification of quantum measurements. We will begin with general POVMs and later focus on the class of measurements with rank-one effects. Before stating the results, let us recall that every quantum measurement can be associated with quantum-classical channel defined as

19 $$\begin{aligned} \mathcal {P}(\rho ) = \sum _i {{\,\mathrm{tr}\,}}(M_i \rho ) |i\rangle \! \langle i|, \end{aligned}$$

where $\{M_i\}_i$ are measurement’s effects and ${{\,\mathrm{tr}\,}}(M_i \rho )$ is the probability of obtaining the *i*-th label.

The following proposition can be seen as a corollary from Theorem 1 as it gives a simple condition when we forbid false negative error. This condition is expressed in terms of inclusion of supports of the measurements’ effects.

### Proposition 2

Let $\mathcal {P}_0$ and $\mathcal {P}_1$ be POVMs with effects $\{M_i\}_{i=1}^m$ and $\{N_i\}_{i=1}^m$ respectively. Then $\mathcal {P}_0$ can be certified against $\mathcal {P}_1$ in the parallel scheme if and only if there exists a pair of effects $M_i$, $N_i$ for which $\mathrm {supp}(M_i) \not \subseteq \mathrm {supp}(N_i)$.

### Proof

Let

20 $$\begin{aligned} M_i = \sum _{k_i} \alpha _{k_i}^i |x_{k_i}^i\rangle \! \langle x_{k_i}^i| \end{aligned}$$

be the spectral decomposition of $M_i$ (where $\alpha _{k_i}^i>0$ for every *k*). Then

21 $$\begin{aligned} \mathcal {P}_0 (\rho ) = \sum _i |i\rangle \! \langle i| {{\,\mathrm{tr}\,}}(M_i \rho ) = \sum _i \sum _{k_i} \alpha _{k_i}^i |i\rangle \! \langle x_{k_i}^i| \rho |x_{k_i}^i\rangle \! \langle i| \end{aligned}$$

and hence the Kraus operators of $\mathcal {P}_0$ are $\left\{ \sqrt{\alpha _{k_i}^i} |i\rangle \! \langle x_{k_i}^i| \right\} _{k_i,i}$. Analogously, the Kraus operators of $\mathcal {P}_1$ are $\left\{ \sqrt{\beta _{k_i}^i} |i\rangle \! \langle y_{k_i}^i| \right\} _{k_i,i}$.

Therefore from Theorem 1 we have that $\mathcal {P}_0$ can be certified against $\mathcal {P}_1$ in the parallel scheme if and only if

22 $$\begin{aligned} \mathrm {span}\left\{ \sqrt{\alpha _{k_i}^i} |i\rangle \! \langle x_{k_i}^i| \right\} _{k_i,i} \not \subseteq \mathrm {span}\left\{ \sqrt{\beta _{k_i}^i} |i\rangle \! \langle y_{k_i}^i| \right\} _{k_i,i}, \end{aligned}$$

that is when there exists a pair of effects $M_i$, $N_i$ for which $\mathrm {supp}(M_i) \not \subseteq \mathrm {supp}(N_i)$. $\square$

The above proposition holds for any pair of quantum measurements. In the case of POVMs with rank-one effects, the above condition can still be simplified to linear independence of vectors. This is stated as the following corollary.

### Corollary 1

Let $\mathcal {P}_0$ and $\mathcal {P}_1$ be measurements with effects $\{ \alpha _i |x_i\rangle \! \langle x_i|\}_{i=1}^m$ and $\{ \beta _i|y_i\rangle \! \langle y_i|\}_{i=1}^m$ for $\alpha _i, \beta _i \in (0, 1]$, respectively. Then $\mathcal {P}_0$ can be certified against $\mathcal {P}_1$ in the parallel scheme if and only if there exists a pair of vectors $|x_i\rangle$, $|y_i\rangle$ which are linearly independent.

While studying the certification of measurements with rank-one effects, one cannot overlook their very important subclass, namely projective von Neumann measurements. These measurements have effects of the form $\{ |u_1\rangle \! \langle u_1|, \ldots , |u_n\rangle \! \langle u_n| \}$, where $\{|u_i\rangle \}_i$ form an orthonormal basis. This class of measurements was studied in^32^, though in a slightly different context. The main result of that work was the expression for minimized probability of the false negative error, where the bound on the false positive error was assumed. In this work, however, we consider the situation when false negative error must be equal zero after sufficiently many uses. Nevertheless, from Corollary 1 we can draw a conclusion that any von Neumann measurement can be certified against some other von Neumann measurement if and only if the measurements are not the same.

### SIC POVMs

Now we proceed to studying the certification of a special class of measurements with rank-one effects, that is symmetric informationally complete (SIC) POVMs^35–38^. We will directly calculate the bounds on the false positive error in the single-shot and parallel certification. We will be using the following notation. The SIC POVM $\mathcal {P}_0$ with effects $\{|x_i\rangle \! \langle x_i|\}_{i=1}^{d^2}$, where $|x_i\rangle \! \langle x_i| = \frac{1}{d} |\phi _i\rangle \! \langle \phi _i|$ and $\Vert |\phi _i\rangle \Vert = 1$, will be associated with the $H_0$ hypothesis. The SIC POVM $\mathcal {P}_1$ corresponding to the alternative $H_1$ hypothesis will have effects $\{|y_i\rangle \! \langle y_i|\}_{i=1}^{d^2}$, where $|y_i\rangle \! \langle y_i| = \frac{1}{d} |\phi _{\pi (i)}\rangle \! \langle \phi _{\pi (i)}|$ and $\pi$ is a permutation of $d^2$ elements. Moreover, the SIC condition assures that $| \langle {\phi _i}|{\phi _{\pi (i)}}\rangle |^2 = \frac{1}{d+1}$ whenever $i \ne \pi (i)$.

#### Remark 1

From Corollary 1 it follows that for a SIC POVMs $\mathcal {P}_0$ can be certified against SIC POVM $\mathcal {P}_1$ in the parallel scheme as long as $\mathcal {P}_0 \ne \mathcal {P}_1$.

Now we are working towards calculating the upper bound on the probability of the false positive error in single-shot certification of SIC POVMs. As the input state we take the maximally entangled state $|\psi \rangle {:}{=}\frac{1}{\sqrt{d}} |{\mathbbm{1}} \rangle \!\rangle$. If the measurement was $\mathcal {P}_0$, then the output state is

23 $$\begin{aligned} \rho _0^{|\psi \rangle } = \left( \mathcal {P}_0 \otimes {\mathbbm{1}} \right) \left( |\psi \rangle \! \langle \psi |\right) = \sum _{i=1}^{d^2} |i\rangle \! \langle i| \otimes \frac{1}{d}(|x_i\rangle \! \langle x_i|)^\top = \sum _{i=1}^{d^2} |i\rangle \! \langle i| \otimes \frac{1}{d^2}(|\phi _i\rangle \! \langle \phi _i|)^\top , \end{aligned}$$

and similarly, if the measurement was $\mathcal {P}_1$, then the output state is

24 $$\begin{aligned} \rho _1^{|\psi \rangle } = \sum _{i=1}^{d^2} |i\rangle \! \langle i| \otimes \frac{1}{d^2}(|\phi _{\pi (i)}\rangle \! \langle \phi _{\pi (i)}|)^\top . \end{aligned}$$

As the output states have block-diagonal structure, we take the measurement effect to be in the block-diagonal form, that is

25 $$\begin{aligned} \Omega _0 {:}{=}\sum _{i=1}^{d^2} |i\rangle \! \langle i| \otimes \Omega _i^\top , \end{aligned}$$

where for every *i* we assume $\Omega _i \perp |\phi _{\pi (i)}\rangle \! \langle \phi _{\pi (i)}|$ to ensure that the probability of the false negative error is equal to zero. We calculate

26 $$\begin{aligned} {{\,\mathrm{tr}\,}}\left( \Omega _0 \rho _0 \right)&= {{\,\mathrm{tr}\,}}\left( \left( \sum _{i=1}^{d^2} |i\rangle \! \langle i| \otimes \Omega _i^\top \right) \left( \sum _{j=1}^{d^2} |j\rangle \! \langle j| \otimes \frac{1}{d^2}(|\phi _j\rangle \! \langle \phi _j|)^\top \right) \right) \\&= {{\,\mathrm{tr}\,}}\left( \sum _{i=1}^{d^2} |i\rangle \! \langle i| \otimes \Omega _i^\top \frac{1}{d^2} (|\phi _i\rangle \! \langle \phi _i|) ^\top \right) =\frac{1}{d^2} \sum _{i=1}^{d^2} \langle \phi _i| \Omega _i |\phi _i\rangle . \end{aligned}$$

Let *k* be the number of fixed points of the permutation $\pi$. Taking $\Omega _i {:}{=}{\mathbbm{1}} - |\phi _{\pi (i)}\rangle \! \langle \phi _{\pi (i)}|$ we obtain

27 $$\begin{aligned} {{\,\mathrm{tr}\,}}\left( \Omega _0 \rho _0 \right)&= \frac{1}{d^2} \sum _{i=1}^{d^2} \langle \phi _i| \Omega _i |\phi _i\rangle = \frac{1}{d^2} \sum _{i=1}^{d^2} \langle \phi _i| \left( {\mathbbm{1}} - |\phi _{\pi (i)}\rangle \! \langle \phi _{\pi (i)}|\right) |\phi _i\rangle \\&= \frac{1}{d^2} \sum _{i=1}^{d^2} \left( 1- |\langle {\phi _i}|{\phi _{\pi (i)}}\rangle |^2 \right) = \frac{1}{d^2} \left( d^2 - k\right) \left( 1- |\langle {\phi _i}|{\phi _{\pi (i)}}\rangle |^2 \right) \\&= \frac{1}{d^2} \left( d^2 - k\right) \left( 1- \frac{1}{d+1} \right) = \frac{d^2 - k}{d^2 + d}. \end{aligned}$$

So far all the calculations were done for some fixed input state (maximally entangled state) and measurement effect $\Omega _0$, which give us actually only the upper bound on the probability of the false positive error. The current choice of $\Omega _i = {\mathbbm{1}} - |\phi _{\pi (i)}\rangle \! \langle \phi _{\pi (i)}|$ seems like a good candidate, but we do not know whether it is possible to find a better one. Using the notation for the probability of the false positive error introduced in Eq. (4) and (2) we can write our bound as

28 $$\begin{aligned} p_1 \le p_1 \left( |\psi \rangle , \Omega _0 \right) = 1-{{\,\mathrm{tr}\,}}( \Omega _0 \rho _0) = \frac{d + k}{d^2+d}. \end{aligned}$$

On top of that, if $\pi$ does not have fixed points, that is when $k=0$, we have $p_1 \le \frac{1}{d+1}$ and the number of steps needed for parallel certification is bounded by $N_\epsilon \ge \left\lceil - \frac{\log \epsilon }{\log (d+1)} \right\rceil$. In the case when the permutation $\pi$ has one fixed point, that is when $k=1$, it holds that $p_1 \le \frac{1}{d}$ and hence the number of steps needed for parallel certification can be bounded by $N_\epsilon \ge \left\lceil - \frac{\log \epsilon }{\log d} \right\rceil$.

### Parallel certification of SIC POVMs

Let us consider a generalization of the results from previous subsection into the parallel scenario. We want to certify SIC POVMs $\mathcal {P}_0$ and $\mathcal {P}_1$ defined as in “SIC POVMs” section, however we assume that we are allowed to use the certified SIC POVM *N* times in parallel. In this setup we associate the $H_0$ hypothesis with the measurement $\mathcal {P}_0^{\otimes N}$, and analogously we associate the $H_1$ hypothesis with the measurement $\mathcal {P}_1^{\otimes N}$. It appears that the upper bound on false positive error is very similar to the upper bound for the single-shot case. Straightforward but lengthy and technical calculations give us

29 $$\begin{aligned} p_1^{\mathbb{P}, N} \le \left( \frac{d + k}{d^2+d}\right) ^N. \end{aligned}$$

The detailed derivation of this bound is relegated to “Supplementary Appendix B”.

## Adaptive certification and Stein setting

So far we were considering only the scheme in which the given channel is used a finite number of times in parallel. In this section we will focus on studying a more general scheme of certification, that is the adaptive certification. In the adaptive scenario, we use the given channel *N* times and between the uses we can perform some processing. It seems natural that the use of adaptive scheme instead of the simple parallel one should improve the certification. Surprisingly, in the case of von Neumann measurements the use of adaptive scheme gives no advantage over the parallel one^18,32^. In other cases it appears that the use of processing is indeed a necessary step towards perfect discrimination^19,20^.

Having the adaptive scheme as a generalization of the parallel one, let us take a step further and take a look into the asymptotic setting. In other words, let us discuss the situation when the number of uses of the certified channel tends to infinity. There are various settings known in the literature concerning asymptotic discrimination, like Stein and Hoeffding settings for asymmetric discrimination, as well as Chernoff and Han-Kobayashi settings for symmetric discrimination. In the context of this work we will discuss only the setting concerning asymmetric discrimination, however a concise introduction to all of these settings can be found e.g. in^33^. Arguably, the most well-known of these is the Hoeffding setting which assumes the bound on the false negative error to be decreasing exponentially, and its area of interest is characterizing the error exponent of probability of false positive error. Adaptive strategies for asymptotic discrimination in Hoeffding setting were recently explored in^21^. In the Stein setting, on the other hand, we assume a constraint on the probability of false positive error and study the error exponent of the false negative error. Let us define a non-asymptotic quantity

30 $$\begin{aligned} \zeta _n (\epsilon ) {:}{=}\sup _{\Omega _0, |\psi \rangle } \left\{ -\frac{1}{n} \log p_2^{\mathbb{A} , n} \left( |\psi \rangle , \Omega _0 \right) : p_1^{\mathbb{A} , n} \left( |\psi \rangle , \Omega _0 \right) \le \epsilon \right\} , \end{aligned}$$

which describes the behavior of probabilities of errors in adaptive discrimination scheme. The probability of false positive error after *n* queries is upper-bounded by some fixed $\epsilon$, and we are interested in studying how quickly the probability of false negative error decreases. Therefore we consider the logarithm of probability of false negative error divided by the number of queries. Finally, a supremum is taken over all possible adaptive strategies, that is we can choose the best input state, final measurement as well as the processings between uses of the certified channel.

Note that in the previous sections we were considering $p_2^{\mathbb{A} , N}$ instead of $p_2^{\mathbb{A} , n}$, which in used in the Stein setting. The aim of this difference is to emphasize that in the Stein setting we study the situation in which the number of uses, *n*, tends to infinity. In contrary, in previous sections we were interested only in the case when the number of uses, *N*, was finite.

Having introduced the non-asymptotic quantity $\zeta _n (\epsilon )$, let us consider the case when the number of queries, *n*, tends to infinity. To do so, we define the upper limit of the Stein exponent as

31 $$\begin{aligned} \overline{\zeta } (\epsilon ) {:}{=}\limsup _{n \rightarrow \infty } \zeta _n (\epsilon ). \end{aligned}$$

Note that when $\overline{\zeta } (\epsilon )$ is finite, then the probability of the false negative error for adaptive certification will not be equal to zero for any finite number of uses *N*. A very useful Remark 19 from^33^ states that $\overline{\zeta }(\epsilon )$ is finite if and only if

32 $$\begin{aligned} \mathrm {supp}\left( (\Phi _0 \otimes {\mathbbm{1}} )(|\psi _\text {ent}\rangle \! \langle \psi _\text {ent}| ) \right) \subseteq \mathrm {supp}\left( (\Phi _1 \otimes {\mathbbm{1}} )(|\psi _\text {ent}\rangle \! \langle \psi _\text {ent}| ) \right) , \end{aligned}$$

where $|\psi _\text {ent}\rangle$ is the maximally entangled state.

Finally, we are in position to express the theorem stating the relation between adaptive and parallel certification.

### Theorem 2

Quantum channel $\Phi _0$ can be certified against quantum channel $\Phi _1$ in the parallel scenario if and only if quantum channel $\Phi _0$ can be certified against quantum channel $\Phi _1$ in the adaptive scenario.

Before presenting the proof of the Theorem we will state a useful lemma, which proof is postponed to “Supplementary Appendix A”.

### Lemma 3

Let $\overline{\zeta }(\epsilon )$ be as in Eq. (31). Then $\overline{\zeta }(\epsilon )$ is finite if and only if $\mathrm {supp}(\Phi _0) \subseteq \mathrm {supp}(\Phi _1)$.

### Proof of Theorem 2

When quantum channel $\Phi _0$ can be certified against the channel $\Phi _1$ in the parallel scenario, then naturally, $\Phi _0$ can be certified against the channel $\Phi _1$ in the adaptive scenario. Therefore it suffices to prove the reverse implication.

Assume that the channel $\Phi _0$ can be certified against $\Phi _1$ in the adaptive scenario. This means that $\overline{\zeta }(\epsilon )$ is infinite. Hence from Lemma 3 it holds that $\mathrm {supp}(\Phi _0) \not \subseteq \mathrm {supp}(\Phi _1)$. Finally, from Theorem 1 we obtain that $\Phi _0$ can be certified against $\Phi _1$ in the parallel scheme. $\square$

Theorem 2 states that if a quantum channel $\Phi _0$ can be certified against $\Phi _1$ in a finite number of queries, then the use of parallel scheme is always sufficient. Therefore it may appear that adaptive certification is of no value. Nevertheless, in some cases it still may be worth using adaptive certification to reduce the number of uses of the certified channel. For example in the case of SIC POVMs the use of adaptive scheme reduces the number of steps significantly^20^. A pair of qutrit SIC POVMs can be discriminated perfectly after two queries in adaptive scenario, therefore they can also be certified. Nevertheless, they cannot be discriminated perfectly after any finite number of queries in parallel. On the other hand, in the case of von Neumann measurements the number of steps is the same no matter which scheme is used^18^.

## Conclusions

As certification of quantum channels is in the NISQ era a task of significant importance, the main aim of this work was to give an insight into this problem from theoretical perspective. Certification was considered as an extension of quantum hypothesis testing, which includes also preparation of an input state and the final measurement. We primarily focused on multiple-shot schemes of certification, that is our areas of interest were mostly parallel and adaptive certification schemes. The parallel scheme consists in certifying tensor products of channels while adaptive scheme is the most general of all scenarios.

We derived a condition when after a finite number of queries in the parallel scenario one can assure that the false negative error will not occur. We pointed a class of channels which allow for excluding false negative error after a finite number of uses in parallel but cannot be discriminated unambiguously. On top of that, having a fixed upper bound on the probability of false positive error, we found a bound on the number of queries needed to make the probability of false positive error no greater than this fixed bound.

Moreover, we took into consideration the most general adaptive certification scheme and studied whether it can improve the certification. It turned out that the use of parallel certification scheme is always sufficient to assure that the false negative error will not occur after a finite number of queries. Nevertheless, the number of queries needed to have the probability of false positive error sufficiently small, may be decreased by using adaptive scheme.

## Supplementary Information

Supplementary Information.
