# Author Correction: Excluding false negative error in certification of quantum channels

**DOI:** 10.1038/s41598-022-10470-y

**Published:** 2022-04-12

**Authors:** Aleksandra Krawiec, Łukasz Pawela, Zbigniew Puchała

**Affiliations:** 1 Institute of Theoretical and Applied Informatics, Polish Academy of Sciences, ul. Bałtycka 5, 44-100 Gliwice, Poland; 2 Faculty of Physics, Astronomy and Applied Computer Science, Jagiellonian University, ul. Łojasiewicza 11, 30-348 Kraków, Poland

Correction to: *Scientific Reports* https://doi.org/10.1038/s41598-021-00444-x, published online 05 November 2021

The Acknowledgements section in the original version of this Article was incomplete.

“This work was supported by the Foundation for Polish Science (FNP) under Grant Number POIR.04.04.00-00-17C1/18-00.”

now reads:

“The project “Near-term quantum computers: Challenges, optimal implementations and applications” under Grant Number POIR.04.04.00-00-17C1/18-00, is carried out within the Team-Net programme of the Foundation for Polish Science co-financed by the European Union under the European Regional Development Fund.”

The original Article has been corrected.

