# Supplementary material for: Excluding false negative error in certification of quantum channels
Source: Sci Rep. 2021 Nov 5;11:21716. doi: 10.1038/s41598-021-00444-x (PMC8571408; doi:10.1038/s41598-021-00444-x)
Supplement: Supplementary file 1 — Supplementary Information. [file 41598_2021_444_MOESM1_ESM.pdf]

EXCLUDING FALSE NEGATIVE ERROR IN CERTIFICATION OF QUANTUM CHANNELS - SUPPLEMENTARY MATERIALS

ALEKSANDRA KRAWIEC[1,*] AND ŁUKASZ PAWELA[1] AND ZBIGNIEW PUCHAŁA[1,2]

[*] E-mail address: akrawiec@iitis.pl

Appendix A. Proofs of lemmas

Proof of Lemma 1. Suppose by contradiction that $\operatorname{supp}\left(\rho_0^{|\psi\rangle}\right) \subseteq \operatorname{supp}\left(\rho_1^{|\psi\rangle}, \ldots, \rho_m^{|\psi\rangle}\right)$, that is

$$\operatorname{span}\left\{(E_i \otimes \mathbb{1})|\psi\rangle\right\}_i \subseteq \operatorname{span}\left\{\left(F_{j_1}^{(1)} \otimes \mathbb{1}\right)|\psi\rangle, \ldots, \left(F_{j_m}^{(m)} \otimes \mathbb{1}\right)|\psi\rangle\right\}_{j_1,\ldots,j_m}. \tag{1}$$

Hence for every i

$$\begin{aligned}(E_i \otimes \mathbb{1})|\psi\rangle &= \sum_{j_1} \beta_{j_1}^{(1)} \left(F_{j_1}^{(1)} \otimes \mathbb{1}\right)|\psi\rangle + \ldots + \sum_{j_m} \beta_{j_m}^{(m)} \left(F_{j_m}^{(m)} \otimes \mathbb{1}\right)|\psi\rangle \\ &= \left(\sum_{j_1} \beta_{j_1}^{(1)} \left(F_{j_1}^{(1)} \otimes \mathbb{1}\right) + \ldots + \sum_{j_m} \beta_{j_m}^{(m)} \left(F_{j_m}^{(m)} \otimes \mathbb{1}\right)\right)|\psi\rangle \\ &= \left(\left(\sum_{j_1} \beta_{j_1}^{(1)} F_{j_1}^{(1)} + \ldots + \sum_{j_m} \beta_{j_m}^{(m)} F_{j_m}^{(m)}\right) \otimes \mathbb{1}\right)|\psi\rangle,\end{aligned} \tag{2}$$

where not all $\beta_{j_k}^{(k)}$ are equal to zero.

As $|\psi\rangle \coloneqq \sum_t \lambda_t |a_t\rangle|b_t\rangle$, we have

$$(E_i \otimes \mathbb{1})|\psi\rangle = \sum_t \lambda_t \left(E_i|a_t\rangle \otimes |b_t\rangle\right) \tag{3}$$

and

$$\begin{aligned}&\left(\left(\sum_{j_1} \beta_{j_1}^{(1)} F_{j_1}^{(1)} + \ldots + \sum_{j_m} \beta_{j_m}^{(m)} F_{j_m}^{(m)}\right) \otimes \mathbb{1}\right)|\psi\rangle \\ &= \sum_t \lambda_t \left(\sum_{j_1} \beta_{j_1}^{(1)} F_{j_1}^{(1)} + \ldots + \sum_{j_m} \beta_{j_m}^{(m)} F_{j_m}^{(m)}\right)|a_t\rangle \otimes |b_t\rangle\end{aligned} \tag{4}$$

As $\{|b_t\rangle\}_t$ is an orthonormal basis, then for every t

$$E_i|a_t\rangle = \left(\sum_{j_1} \beta_{j_1}^{(1)} F_{j_1}^{(1)} + \ldots + \sum_{j_m} \beta_{j_m}^{(m)} F_{j_m}^{(m)}\right) |a_t\rangle \tag{5}$$

and hence

$$E_i = \sum_{j_1} \beta_{j_1}^{(1)} F_{j_1}^{(1)} + \ldots + \sum_{j_m} \beta_{j_m}^{(m)} F_{j_m}^{(m)}. \tag{6}$$

Therefore

$$\operatorname{span}\{E_i\}_i \subseteq \operatorname{span}\left\{F_{j_1}^{(1)}, \ldots, F_{j_m}^{(m)}\right\}_{j_1,\ldots,j_m} \tag{7}$$

which implies that $\operatorname{supp}(\Phi_0) \subseteq \operatorname{supp}(\Phi_1, \ldots, \Phi_m)$.

Finally, from the law of contraposition we obtain that if $\operatorname{supp}(\Phi_0) \not\subseteq \operatorname{supp}(\Phi_1, \ldots, \Phi_m)$, then $\operatorname{supp}\left(\rho_0^{|\psi\rangle}\right) \not\subseteq \operatorname{supp}\left(\rho_1^{|\psi\rangle}, \ldots, \rho_m^{|\psi\rangle}\right)$. □

Proof of Lemma 2. Assume by contradiction that $\operatorname{supp}(\Phi_0) \subseteq \operatorname{supp}(\Phi_1, \ldots \Phi_m)$, that is

$$\operatorname{span}\{E_i\}_i \subseteq \operatorname{span}\left\{F_{j_1}^{(1)}, \ldots, F_{j_m}^{(m)}\right\}_{j_1,\ldots,j_m} \tag{8}$$

To simplify the notation, without loss of generality we define $\operatorname{span}\left\{F_{j_1}^{(1)}, \ldots, F_{j_m}^{(m)}\right\}_{j_1,\ldots,j_m} =: \operatorname{span}\{K_l\}_l$. Hence for every natural number N it also holds that

$$\operatorname{span}\{E_{i_1} \otimes \ldots \otimes E_{i_N}\}_{i_1,\ldots,i_N} \subseteq \operatorname{span}\{K_{l_1} \otimes \ldots \otimes K_{l_N}\}_{l_1,\ldots,l_N}. \tag{9}$$

Thus for every $i_1, \ldots, i_N$ we have that

$$E_{i_1} \otimes \ldots \otimes E_{i_N} = \sum_{l_1,\ldots,l_N} \beta_{l_1,\ldots,l_N} K_{l_1} \otimes \ldots \otimes K_{l_N}, \tag{10}$$

where not all $\beta_{l_1,\ldots,l_N}$ are equal to zero. Therefore for every $i_1, \ldots, i_N$ and input state $|\psi\rangle$, it also holds that

$$\begin{aligned}((E_{i_1} \otimes \ldots \otimes E_{i_N}) \otimes \mathbb{1})|\psi\rangle &= \left(\left(\sum_{l_1,\ldots,l_N} \beta_{l_1,\ldots,l_N} K_{l_1} \otimes \ldots \otimes K_{l_N}\right) \otimes \mathbb{1}\right)|\psi\rangle \\ &= \sum_{l_1,\ldots,l_N} \beta_{l_1,\ldots,l_N} ((K_{l_1} \otimes \ldots \otimes K_{l_N}) \otimes \mathbb{1})|\psi\rangle\end{aligned} \tag{11}$$

and hence

$$\operatorname{span}\{((E_{i_1} \otimes \ldots \otimes E_{i_N}) \otimes \mathbb{1})|\psi\rangle\}_{i_1,\ldots,i_N} \subseteq \operatorname{span}\{((K_{l_1} \otimes \ldots \otimes K_{l_N}) \otimes \mathbb{1})|\psi\rangle\}_{l_1,\ldots,l_N}. \tag{12}$$

The above can be rewritten as

$$\operatorname{supp}\left(\sigma_0^{N,|\psi\rangle}\right) \subseteq \operatorname{supp}\left(\sigma_1^{N,|\psi\rangle}, \ldots, \sigma_m^{N,|\psi\rangle}\right), \quad N \in \mathbb{N}. \tag{13}$$

Eventually, by the law of contraposition we obtain that if for some natural number N and an input state $|\psi\rangle$ it holds that

$$\operatorname{supp}\left(\sigma_0^{N,|\psi\rangle}\right) \not\subseteq \operatorname{supp}\left(\sigma_1^{N,|\psi\rangle}, \ldots, \sigma_m^{N,|\psi\rangle}\right), \tag{14}$$

then $\operatorname{supp}(\Phi_0) \nsubseteq \operatorname{supp}(\Phi_1, \dots \Phi_m)$. □

Proof of Lemma 3. ($\Longrightarrow$) Let $|\psi_{\text{ent}}\rangle$ be the maximally entangled state. When $\overline{\zeta}(\epsilon) < \infty$, then from Remark 19 from [1] we have that

$$\operatorname{supp}\left((\Phi_0 \otimes \mathbb{1})(|\psi_{\text{ent}}\rangle\langle\psi_{\text{ent}}|)\right) \subseteq \operatorname{supp}\left((\Phi_1 \otimes \mathbb{1})(|\psi_{\text{ent}}\rangle\langle\psi_{\text{ent}}|)\right). \tag{15}$$

From Lemma 1, (as the maximally entangled state has full Schmidt rank), the above implies that $\operatorname{supp}(\Phi_0) \subseteq \operatorname{supp}(\Phi_1)$.

($\Longleftarrow$) Now we assume that $\operatorname{supp}(\Phi_0) \subseteq \operatorname{supp}(\Phi_1)$. From Lemma 2, this implies that for every natural number N and every input state $|\psi\rangle$ it holds that

$$\operatorname{supp}\left((\Phi_0^{\otimes N} \otimes \mathbb{1})(|\psi\rangle\langle\psi|)\right) \subseteq \operatorname{supp}\left((\Phi_1^{\otimes N} \otimes \mathbb{1})(|\psi\rangle\langle\psi|)\right). \tag{16}$$

Taking $N = 1$ and $|\psi\rangle = |\psi_{\text{ent}}\rangle$ we obtain

$$\operatorname{supp}\left((\Phi_0 \otimes \mathbb{1})(|\psi_{\text{ent}}\rangle\langle\psi_{\text{ent}}|)\right) \subseteq \operatorname{supp}\left((\Phi_1 \otimes \mathbb{1})(|\psi_{\text{ent}}\rangle\langle\psi_{\text{ent}}|)\right). \tag{17}$$

Therefore from Remark 19 from [1] we obtain that $\overline{\zeta}(\epsilon) < \infty$. □

Appendix B. Derivation of Eq. (29)

Let $\mathcal{P}_0$ and $\mathcal{P}_1$ be as defined in Subsection "SIC POVMs". We consider the scenario where the certified measurement is used N times in parallel. To calculate the bound on the parallel certification we will take particular choices of an input state and a final measurement. As for the input state, we will take the maximally entangled state, similarly as it was in the single-shot case. Applying tensor product of the SIC POVMs on the input state we obtain the output states either

$$\begin{aligned} \sigma_0^{N,|\psi\rangle} &= \frac{1}{d^N}\left(\mathcal{P}_0 \otimes \ldots \otimes \mathcal{P}_0 \otimes \mathbb{1}\right)\left(|\mathbb{1}\rangle\!\rangle\langle\!\langle\mathbb{1}|\right) \\ &= \frac{1}{d^N}\sum_{i_1,\ldots,i_N=1}^{d^2} |i_1 \cdots i_N\rangle\langle i_1 \cdots i_N| \otimes \frac{1}{d^N}\left(|\phi_{i_1}\cdots\phi_{i_N}\rangle\langle\phi_{i_1}\cdots\phi_{i_N}|\right)^\top \\ &= \frac{1}{d^{2N}}\sum_{i_1,\ldots,i_N=1}^{d^2} |i_1 \cdots i_N\rangle\langle i_1 \cdots i_N| \otimes \left(|\phi_{i_1}\cdots\phi_{i_N}\rangle\langle\phi_{i_1}\cdots\phi_{i_N}|\right)^\top \end{aligned} \tag{18}$$

if the measurement was $\mathcal{P}_0$, or

$$\sigma_1^{N,|\psi\rangle} = \frac{1}{d^{2N}}\sum_{i_1,\ldots,i_N=1}^{d^2} |i_1 \cdots i_N\rangle\langle i_1 \cdots i_N| \otimes \left(|\phi_{\pi(i_1)}\cdots\phi_{\pi(i_N)}\rangle\langle\phi_{\pi(i_1)}\cdots\phi_{\pi(i_N)}|\right)^\top \tag{19}$$

if the measurement was $\mathcal{P}_1$. Similarly to the single-shot scenario, we take the measurement effect with block-diagonal structure, that is

$$\Omega_0 \coloneqq \sum_{i_1,\ldots,i_N=1}^{d^2} |i_1 \cdots i_N\rangle\langle i_1 \cdots i_N| \otimes \Omega_{i_1\cdots i_N}^\top \tag{20}$$

where we require $\Omega_{i_1\cdots i_N} \perp |\phi_{\pi(i_1)}\cdots\phi_{\pi(i_N)}\rangle\langle\phi_{\pi(i_1)}\cdots\phi_{\pi(i_N)}|$ to make sure that the false negative error will be equal zero.

We calculate

(21)

$$\begin{aligned}
\operatorname{tr}\left(\Omega_0\sigma_0^{N,|\psi\rangle}\right) &= \operatorname{tr}\left(\left(\sum_{i_1,\ldots,i_N=1}^{d^2}|i_1\cdots i_N\rangle\langle i_1\cdots i_N|\otimes\Omega_{i_1\cdots i_N}^\top\right)\right.\\
&\quad\left.\left(\frac{1}{d^{2N}}\sum_{k_1,\ldots,k_N=1}^{d^2}|k_1\cdots k_N\rangle\langle k_1\cdots k_N|\otimes\left(|\phi_{k_1}\cdots\phi_{k_N}\rangle\langle\phi_{k_1}\cdots\phi_{k_N}|\right)^\top\right)\right)\\
&= \frac{1}{d^{2N}}\sum_{i_1,\ldots,i_N=1}^{d^2}\sum_{k_1,\ldots,k_N=1}^{d^2}\operatorname{tr}\left(\left(|i_1\cdots i_N\rangle\langle i_1\cdots i_N|\otimes\Omega_{i_1\cdots i_N}^\top\right)\right.\\
&\quad\left.\left(|k_1\cdots k_N\rangle\langle k_1\cdots k_N|\otimes\left(|\phi_{k_1}\cdots\phi_{k_N}\rangle\langle\phi_{k_1}\cdots\phi_{k_N}|\right)^\top\right)\right)\\
&= \frac{1}{d^{2N}}\sum_{i_1,\ldots,i_N=1}^{d^2}\sum_{k_1,\ldots,k_N=1}^{d^2}\operatorname{tr}\left(|i_1\cdots i_N\rangle\langle i_1\cdots i_N||k_1\cdots k_N\rangle\langle k_1\cdots k_N|\right.\\
&\quad\left.\otimes\Omega_{i_1\cdots i_N}^\top\left(|\phi_{k_1}\cdots\phi_{k_N}\rangle\langle\phi_{k_1}\cdots\phi_{k_N}|\right)^\top\right)\\
&= \frac{1}{d^{2N}}\sum_{i_1,\ldots,i_N=1}^{d^2}\operatorname{tr}\left(|i_1\cdots i_N\rangle\langle i_1\cdots i_N|\otimes\Omega_{i_1\cdots i_N}^\top\left(|\phi_{i_1}\cdots\phi_{i_N}\rangle\langle\phi_{i_1}\cdots\phi_{i_N}|\right)^\top\right)\\
&= \frac{1}{d^{2N}}\sum_{i_1,\ldots,i_N=1}^{d^2}\operatorname{tr}\left(\Omega_{i_1\cdots i_N}|\phi_{i_1}\cdots\phi_{i_N}\rangle\langle\phi_{i_1}\cdots\phi_{i_N}|\right).
\end{aligned}$$

There are many possible choices of such $\Omega_{i_1\cdots i_N}$ which fulfill the condition $\Omega_{i_1\cdots i_N}\perp|\phi_{\pi(i_1)}\cdots\phi_{\pi(i_N)}\rangle\langle\phi_{\pi(i_1)}\cdots\phi_{\pi(i_N)}|$, but for the time being we will take the one defined as follows

$$\Omega_{i_1\cdots i_N} := \mathbb{1}-|\phi_{\pi(i_1)}\cdots\phi_{\pi(i_N)}\rangle\langle\phi_{\pi(i_1)}\cdots\phi_{\pi(i_N)}|. \tag{22}$$

This choice of measurement effect may not appear optimal in general, but it is suitable for calculations due to its concise form. Therefore

(23)

$$\begin{aligned}
\operatorname{tr}\left(\Omega_0\sigma_0^{N,|\psi\rangle}\right) &= \frac{1}{d^{2N}}\sum_{i_1,\ldots,i_N=1}^{d^2}\operatorname{tr}\left(\left(\mathbb{1}-|\phi_{\pi(i_1)}\cdots\phi_{\pi(i_N)}\rangle\langle\phi_{\pi(i_1)}\cdots\phi_{\pi(i_N)}|\right)|\phi_{i_1}\cdots\phi_{i_N}\rangle\langle\phi_{i_1}\cdots\phi_{i_N}|\right)\\
&= \frac{1}{d^{2N}}\sum_{i_1,\ldots,i_N=1}^{d^2}\left(1-\left|\langle\phi_{i_1}\cdots\phi_{i_N}|\phi_{\pi(i_1)}\cdots\phi_{\pi(i_N)}\rangle\right|^2\right)\\
&= 1-\frac{1}{d^{2N}}\sum_{i_1,\ldots,i_N=1}^{d^2}\left|\langle\phi_{i_1}\cdots\phi_{i_N}|\phi_{\pi(i_1)}\cdots\phi_{\pi(i_N)}\rangle\right|^2.
\end{aligned}$$

Therefore we have

$$p_1^{\mathbb{P},N}\left(|\psi\rangle,\Omega_0\right)=1-\operatorname{tr}\left(\Omega_0\sigma_0^{N,|\psi\rangle}\right)=\frac{1}{d^{2N}}\sum_{i_1,\ldots,i_N=1}^{d^2}\left|\langle\phi_{i_1}\cdots\phi_{i_N}|\phi_{\pi(i_1)}\cdots\phi_{\pi(i_N)}\rangle\right|^2. \tag{24}$$

To get the exact upper bound we need to calculate the sum, that is to explain that

$$\sum_{i_1,\ldots,i_N=1}^{d^2}\left|\langle\phi_{i_1}\cdots\phi_{i_N}|\phi_{\pi(i_1)}\cdots\phi_{\pi(i_N)}\rangle\right|^2=\sum_{s=0}^{N}\binom{N}{N-s}k^{N-s}(d^2-k)^s\frac{1}{(d+1)^s}. \tag{25}$$

First, note that

$$\left|\langle\phi_{i_1}\cdots\phi_{i_N}|\phi_{\pi(i_1)}\cdots\phi_{\pi(i_N)}\rangle\right|^2=\left|\langle\phi_{i_1}|\phi_{\pi(i_1)}\rangle\cdots\langle\phi_{i_N}|\phi_{\pi(i_N)}\rangle\right|^2=\frac{1}{(d+1)^s}, \tag{26}$$

where $s:=|\{i_l:\ i_l\neq\pi(i_l)\}|$. In other words, every time we encounter a fixed point of the permutation we have a factor $\langle\phi_{i_j}|\phi_{\pi(i_j)}\rangle$ which is equal one. Let us now focus on consecutive factors of the right hand site of the Eq. (25). The factor $\binom{N}{N-s}$ corresponds to choosing $N-s$ elements for which $\langle\phi_{i_j}|\phi_{\pi(i_j)}\rangle=1$. Then, on each of those elements there can be one of k elements (as k stands for the number of fixed points of the permutation π), therefore k^{N-s}. Then, on the remaining s elements there can one of d^2-k values which are not fixed points of the permutation, hence we obtain $(d^2-k)^s$. Further calculations reveal the concise expression for the upper bound on the probability of false negative error, that is

$$p_1^{\mathbb{P},N}\left(|\psi\rangle,\Omega_0\right)=\frac{1}{d^{2N}}\sum_{s=0}^{N}\binom{N}{N-s}k^{N-s}(d^2-k)^s\frac{1}{(d+1)^s}=\left(\frac{d+k}{d^2+d}\right)^N. \tag{27}$$

In the case of permutation π having no fixed points, that is when $k=0$, the above bound simplifies to $p_1^{\mathbb{P},N}\leq\left(\frac{1}{d+1}\right)^N$.

References

[1] M. M. Wilde, M. Berta, C. Hirche, and E. Kaur, “Amortized channel divergence for asymptotic quantum channel discrimination,” *Letters in Mathematical Physics*, vol. 110, no. 8, pp. 2277–2336, 2020.
